# A complete blood count-based multivariate model for predicting the recovery of patients with moderate COVID-19: a retrospective study

**DOI:** 10.1038/s41598-022-23285-8

**Published:** 2022-10-29

**Authors:** Yiting Wang, Xuewen Li, Jiancheng Xu, Qi Zhou

**Affiliations:** 1 Department of Laboratory Medicine, First Hospital of Jilin University, 1 Xinmin Street, Changchun, 130021 China; 2 Department of Pediatrics, First Hospital of Jilin University, 1 Xinmin Street, Changchun, 130021 China

**Keywords:** Viral infection, Nomograms, Epidemiology, Prognostic markers

## Abstract

Many resource-limited countries need an efficient and convenient method to assess disease progression in patients with coronavirus disease 2019 (COVID-19). This study developed and validated a complete blood count-based multivariate model for predicting the recovery of patients with moderate COVID-19. We collected the clinical data and laboratory test results of 86 patients with moderate COVID-19. These data were categorized into two subgroups depending on the laboratory test time. Univariate logistic regression and covariance diagnosis were used to screen for independent factors, and multifactorial logistic regression was used for model building. Data from 38 patients at another hospital were collected for external verification of the model. Basophils (OR 6.372; 95% CI 3.284–12.363), mean corpuscular volume (OR 1.244; 95% CI 1.088–1.422), red blood cell distribution width (OR 2.585; 95% CI 1.261–5.297), and platelet distribution width (OR 1.559; 95% CI 1.154–2.108) could be combined to predict recovery of patients with moderate COVID-19. The ROC curve showed that the model has good discrimination. The calibration curve showed that the model was well-fitted. The DCA showed that the model is clinically useful. Small increases in the above parameters within the normal range suggest an improvement in patients with moderate COVID-19.

## Introduction

The coronavirus disease 2019 (COVID-19) began to spread globally in December 2019, posing a serious pandemic and threat to human health^1^. As of March 21, 2022, there have been over 469 million confirmed cases of COVID-19 worldwide and over 6.07 million deaths^2^. Currently, the severe acute respiratory syndrome coronavirus 2 (SARS-CoV-2) that causes the COVID-19 disease is detected using reverse transcription-polymerase chain reaction (RT-PCR)^3^. Although this method has reasonable specificity and sensitivity, it requires specialized equipment, reagents, and personnel training^4^. It also takes a relatively long time to get results and is costly^5^. A positive RT-PCR test result indicates a confirmed COVID-19 case^6^. Some countries discharge inpatients with COVID-19 if two RT-PCR tests are negative more than 24 h apart^7^. However, the discharge criteria vary widely among countries and some do not have specific discharge criteria^8^. Thus, with limited time and human and material resources, there is an urgent need for a rapid, simple, and affordable method of monitoring illness progression in patients and determining when they can be discharged from the hospital.

Most previous studies focused on the imaging examination and clinical symptoms of patients with COVID-19, which frequently required additional expenses and energy, burdened patients physically or financially, and lacked a fast and cost-effective method to predict patients’ disease process^9,10^. Blood closely interacted with various tissues and cells in the body and it could provide a wide range of information^11^. A complete blood count (CBC) was the most common test done in clinical practice on hospitalized patients^12^. It was a simple, quick, and low-cost test^13^. COVID-19 had been shown to affect the blood circulatory system, and obvious and persistent changes in blood cells could be detected during the infection^14,15^. Many studies done on patients with mild and severe COVID-19, and found that blood cell changes correlate strongly with the severity of COVID-19^16–18^. But few studies had been performed on patients with moderate cases. Of the 72,314 local COVID-19 cases reported by the Chinese Center for Disease Control and Prevention, a majority (81%) had mild or moderate cases^19^. According to the World Health Organization, as of March 21, 2022, the percentage of deaths following COVID-19 infection was approximately 1.3% and the rate of patients treated and discharged was about 86.6%^2^. Therefore, it is of more practical significance to assess the progression of disease in patients with moderate COVID-19 and find the factors related to the improvement of patients’ conditions. This study will conduct a retrospective multicenter study to analyze the role of a CBC test in the rehabilitation of patients with moderate COVID-19 for the first time. On this basis, an efficient and convenient multivariable combination model will be developed to predict patient recovery.

## Methods

### Study design and patient population

We retrospectively analyzed data of 127 patients with COVID-19 from the electronic medical record systems of Changchun Chinese Medicine Hospital and Siping Infectious Diseases Hospital from January 2020 to March 2021. The data included gender, age, comorbidities, clinical symptoms, length of hospitalization, and results of multiple laboratory tests after admission. All patients in the study had data on admission, multiple times after admission, turning negative and after discharge. The inclusion and discharge criteria were found in the Diagnosis and treatment protocol for novel coronavirus pneumonia (Trial Version 7)^20^. Inclusion criteria were as follows: Mild cases had mild clinical symptoms and no signs of pneumonia on imaging. Moderate cases had a fever and respiratory symptoms with imaging findings of pneumonia. Cases meeting any of the following criteria were defined as severe cases: Respiratory distress (respiratory rate, ≥ 30 breaths/min); oxygen saturation ≤ 93% at rest; arterial oxygen partial pressure/fraction of inspired oxygen ≤ 300 mmHg. Lung imaging indicated that the lesions progressed significantly within 24–48 h, and patients with lung lesions occupying > 50% of the lung were treated according to management protocols for severe cases. Cases meeting any of the following criteria were defined as critical cases: Respiratory failure and requirement of mechanical ventilation; shock; combination with failure of other organs that required care in the intensive care unit. The mild, severe, or critical cases were excluded according to the criteria. Discharge criteria were as follows: Body temperature had been back to normal for more than three days; respiratory symptoms improved obviously; pulmonary imaging showed obvious absorption of inflammation; nucleic acid tests were negative twice consecutively on respiratory tract samples such as sputum and nasopharyngeal swabs (sampling interval being at least 24 h). The verification cohort consisted of 38 patients with moderate COVID-19 in Changchun Infectious Disease Hospital from January to March 2020. The data were divided into two subgroups according to the laboratory test time. The first test results after admission went into the early onset group, usually within 1–3 days after admission. The first test results within 3 days before discharge went into the turning negative group. The study was conducted in accordance with the Declaration of Helsinki (as revised in 2013). The study was approved by the Ethics Committee of three hospitals (the Ethics Committee of Changchun Infectious Disease Hospital, No. 2020-001; the Ethics Committee of Changchun Chinese Medicine Hospital, No. 2021-005; the Ethics Committee of Siping Infectious Disease Hospital, No. 2020-001). The requirement for written informed consent was waived due to the study’s retrospective nature by the ethics committees (the Ethics Committee of Changchun Infectious Disease Hospital, the Ethics Committee of Changchun Chinese Medicine Hospital, and the Ethics Committee of Siping Infectious Disease Hospital).

### Data collection

COVID-19 results were confirmed through the Changchun Center for Disease Control and Prevention, Siping Center for Disease Control and Prevention, or Jilin Center for Disease Control and Prevention. Laboratory tests included hematology and biochemical tests. The biochemical and hematology equipment used at Changchun Chinese Medicine Hospital were B-S800M (Mindray Biomedical Electronics Corp., Shenzhen, China) and BC-5390 (Mindray Biomedical Electronics Corp., Shenzhen, China). The biochemical and hematology equipment used at Siping Infectious Disease Hospital were Pointcare M3i (Mnchip Technology Corp., Tianjin, China) and ABX Pentra XL 80 (Horiba Medical, Montpellier, France). The biochemical and hematology equipment used at Changchun Infectious Disease Hospital were CS-T300 (Dirui Industrial Corp., Changchun, China) and DF53 (Dymind Biotechnology Corp., Shenzhen, China). The instruments underwent rigorous quality control testing. The three laboratories that participated in this study all passed the external quality assessment and proficiency certification of the Jilin Clinical Laboratory Center. All physicians, technicians, and nurses in this study received uniform training from the Health Commission of Jilin Province. The National Health Commission of the People’s Republic of China had issued the reference interval standards for common biochemical analyte and blood cell analysis of Chinese adults^21,22^. The reference intervals used by three hospitals followed the standards. There was no influence of different instruments on the test results.

### Statistical analysis

The Kolmogorov–Smirnov test was used to test the normality of the quantitative data. The quantitative data with normal distribution were compared using the independent-samples *t-*test and expressed as $\overline{x }$ ± *s* [Average ± standard deviation]; and quantitative data with non-normal distribution were compared using the Mann–Whitney *U* test and expressed as *M* (*P*_25_, *P*_75_) [Median (25th percentile, 75th percentile)]. The qualitative data were compared using the chi-squared test or Fisher’s exact test and expressed as *n* (frequency). Three methods were used to screen for independent factors for the recovery of patients with moderate COVID-19. Variables with *P* values greater than or equal to 0.05 were excluded by the univariate logistic regression analysis. The Spearman correlation was used to determine whether there was a significant correlation. Collinearity diagnostics was used to screen the variables to avoid possible multicollinearity of the model. In general, a variance inflation factor (VIF) greater than 10 and tolerance less than 0.2 indicated possible multicollinearity between the independent variables and were excluded. Finally, the multifactorial logistic regression included the variables that met the requirements. The multivariate model was fitted using the Backward: Likelihood Ratio method to calculate the odds ratio (OR) and 95% confidence interval (CI) for each variable. The combined model was presented as a nomogram. The receiver operating characteristic (ROC) curve was used to assess the predictive model discrimination and calculate the area under the curve (AUC) and the 95% CI. A *P* value less than 0.05 was considered statistically significant. The model’s goodness-of-fit was assessed using the calibration curve and a *P* value greater than 0.05 was considered a satisfactory fit. The clinical usefulness of the model was evaluated using a decision curve analysis (DCA). Stata 15, GraphPad Prism 8, and SPSS 23.0 were used for data analysis and graphical plotting.

### Ethics approval and consent to participate

The Declaration of Helsinki conducted the study (as revised in 2013). The study was approved by the Ethics Committee of Changchun Infectious Disease Hospital (No. 2020-001), the Ethics Committee of Changchun Chinese Medicine Hospital (No. 2021-005), and the Ethics Committee of Siping Infectious Disease Hospital (No. 2020-001). The requirement for written informed consent was waived due to the study’s retrospective nature by the ethics committees.

## Results

### Clinical and laboratory characteristics of patients with moderate COVID-19

**Figure 1:**
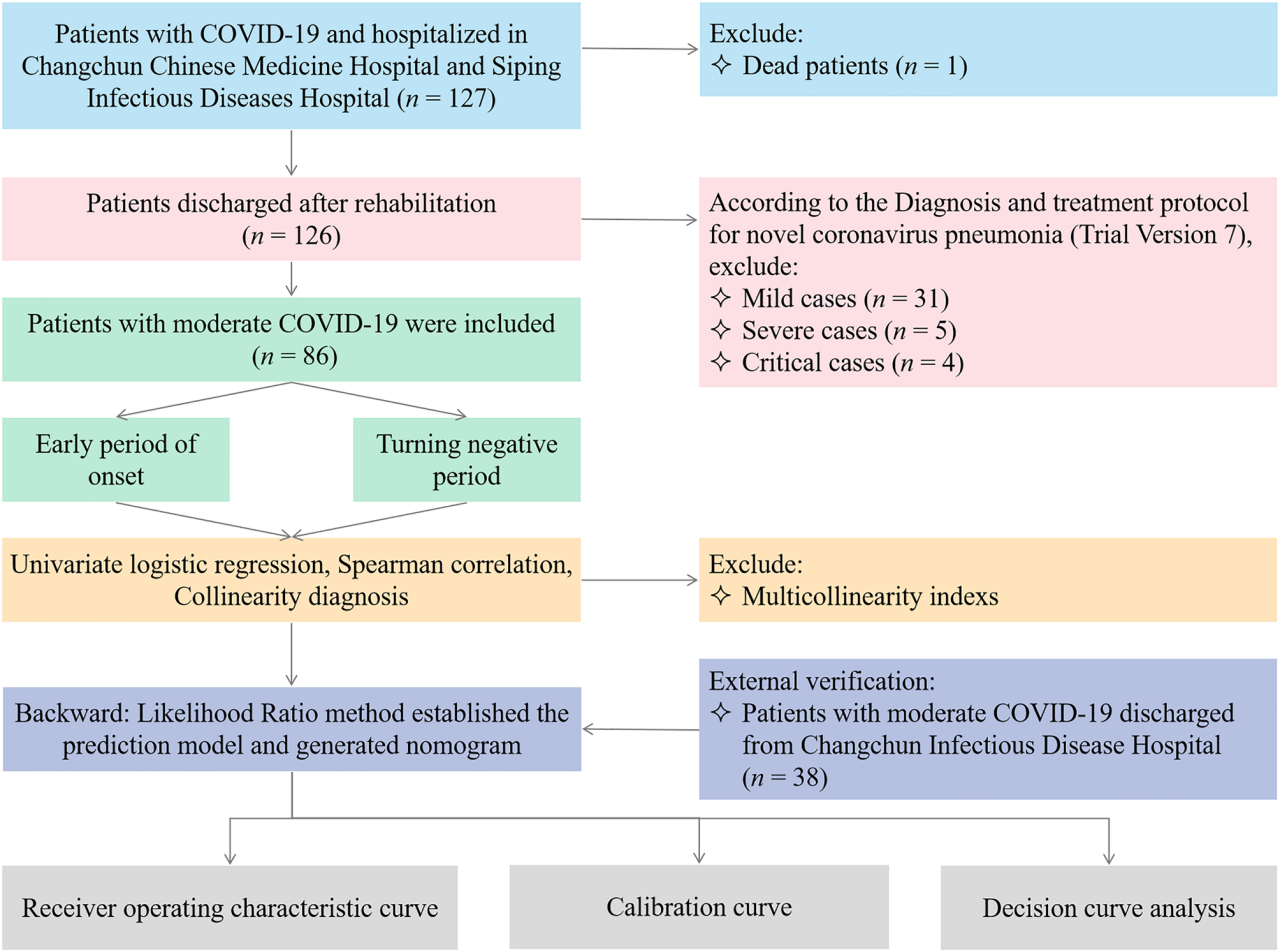
The flowchart of this study.

**Figure 2:**
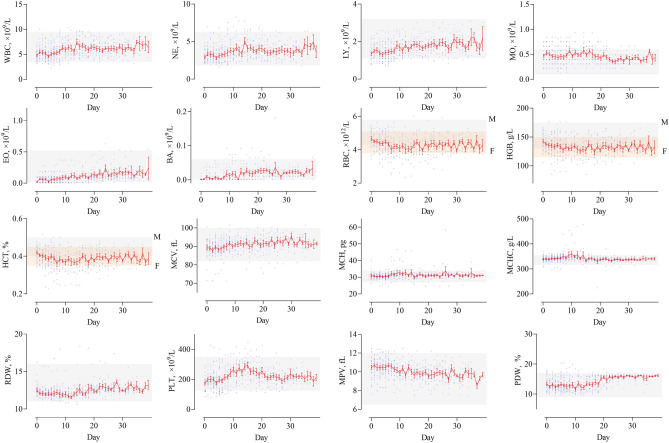
Changes of blood cell parameters with time in patients with COVID-19 during hospitalization and after discharge. *M* male, *F* female. The shaded parts were the reference intervals of the tests.

Among 127 patients with COVID-19, those excluded were one patient who died, 31 patients with mild cases, 5 patients with severe cases, and 4 patients with critical cases. A total of 86 patients with moderate COVID-19 were finally included and the patient selection flowchart was shown in Fig. 1. Their mean age was 53 years, their mean hospital stay was 20 days, the most common comorbidity was cardiovascular disease (25.6%), and the most common clinical symptom was cough (41.9%). Men made up 43% of the cohort. There were no statistically significant gender differences in the length of hospital stays, age, comorbidities (except cerebrovascular disease), clinical symptoms, and medication use (*P* > 0.05). The clinical characteristics of these patients were shown in Table 1. The red blood cell, hemoglobin, hematocrit, platelet count (PLT), mean platelet volume (MPV), creatinine, total protein (TP), albumin (ALB), and potassium (K) of the patients were normally distributed (*P* > 0.05). The independent samples *t-*test and Mann–Whitney *U* test showed white blood cells (WBC), neutrophil count (NE), lymphocyte count (LY), eosinophils (EO), basophils (BA), mean corpuscular volume (MCV), mean corpuscular hemoglobin concentration, red blood cell distribution width (RDW), PLT, MPV, platelet distribution width (PDW), glucose, Cr, urea, carbon dioxide combining power, TP, ALB, aspartate aminotransferase, alkaline phosphatase, γ-glutamyl transpeptidase, sodium, K, and chloride showed statistically significant differences between the early onset and turning negative data (*P* < 0.05), as detailed in Table 2. We collected longitudinal changes in CBC in these patients after hospitalization and rehabilitation discharge. Most of the changes in CBC indicators were within the reference ranges and the trajectory of each indicator over time was shown in Fig. 2.

**Table 1:** Clinical characteristics of patients with moderate COVID-19.

Characteristics	Total (*n* = 86)	Male (*n* = 37)	Female (*n* = 49)	*t*/χ^2^ value	*P* value
Hospitalized time, day, $\overline{x }$± *s*	20.27 ± 5.95	19.97 ± 4.58	20.49 ± 6.85	− 0.397	0.693
Age, year, $\overline{x }$± *s*	53.08 ± 16.74	51.54 ± 16.65	54.24 ± 16.88	− 0.740	0.461
**Any comorbidities *n* (%)**					
Endocrine system disease	10 (11.6)	2 (5.4)	8 (16.3)	–	0.177
Cardiovascular disease	22 (25.6)	10 (27.0)	12 (24.5)	0.071	0.789
Cerebrovascular disease	5 (5.8)	5 (13.5)*	–	–	0.013
Others	9 (10.5)	4 (10.8)	5 (10.2)	–	1.000
**Chief complaint *n* (%)**					
Cough	36 (41.9)	12 (32.4)	24 (49.0)	2.372	0.124
Fever	15 (17.4)	6 (16.2)	9 (18.4)	0.068	0.795
Fatigue	7 (8.1)	3 (8.1)	4 (8.2)	–	1.000
Myalgia	4 (4.7)	1 (2.7)	3 (6.1)	–	0.631
Pleuritic chest pain	3 (3.5)	1 (2.7)	2 (4.1)	–	1.000
Others	4 (4.7)	1 (2.7)	3 (6.1)	–	0.631
**Drug use *n* (%)**					
Traditional Chinese medicine	80 (93.0)	35 (94.6)	45 (91.8)	–	0.696
Antiviral drugs	55 (64.0)	23 (62.2)	32 (65.3)	0.090	0.764
Anti-inflammatory drugs	12 (14.0)	4 (10.8)	8 (16.3)	0.534	0.465
Others	36 (41.9)	15 (40.5)	21 (42.9)	0.046	0.829

$\overline{x }$± *s* Average ± standard deviation.

*Significant difference between the two groups, *P* < 0.05.

**Table 2:** Laboratory characteristics of patients with moderate COVID-19.

Analytes	Early period of onset	Turning negative period	*t*/*Z* value	*P* value
**Leukocyte parameters**				
WBC, × 10^9^/L, *M* (*P*_25_, *P*_75_)	5.10 (4.10, 5.93)*	6.49 (5.31, 7.96)	− 5.184	< 0.001
NE, × 10^9^/L, *M* (*P*_25_, *P*_75_)	3.00 (2.30, 3.83)*	3.90 (3.00, 4.93)	− 4.138	< 0.001
LY, × 10^9^/L, *M* (*P*_25_, *P*_75_)	1.30 (1.00, 1.80)*	1.90 (1.50, 2.20)	− 5.072	< 0.001
MO, × 10^9^/L, *M* (*P*_25_, *P*_75_)	0.50 (0.40, 0.60)	0.40 (0.30, 0.50)	− 1.936	0.053
EO, × 10^9^/L, *M* (*P*_25_, *P*_75_)	0.00 (0.00, 0.10)*	0.10 (0.06, 0.19)	− 6.260	< 0.001
BA, × 10^9^/L, *M* (*P*_25_, *P*_75_)	0.00 (0.00, 0.00)*	0.02 (0.02, 0.04)	− 10.167	< 0.001
**Erythrocyte parameters**				
RBC, × 10^12^/L, $\overline{x }$ ± *s*	4.48 ± 0.54	4.40 ± 0.55	0.971	0.333
HGB, g/L, $\overline{x }$ ± *s*	137.30 ± 16.14	135.26 ± 14.48	0.875	0.383
HCT, L/L, $\overline{x }$ ± *s*	0.40 ± 0.05	0.40 ± 0.04	0.278	0.781
MCV, fL, *M* (*P*_25_, *P*_75_)	89.50 (86.90, 92.33)*	91.70 (90.08, 94.45)	− 3.827	< 0.001
MCH, pg, *M* (*P*_25_, *P*_75_)	30.65 (29.50, 31.73)	31.10 (30.00, 32.00)	− 1.202	0.229
MCHC, g/L, *M* (*P*_25_, *P*_75_)	339.00 (334.00, 347.25)*	337.00 (333.00, 342.25)	− 2.307	0.021
RDW, %, *M* (*P*_25_, *P*_75_)	12.05 (11.60, 12.53)*	12.40 (12.08, 12.95)	− 2.914	0.004
**Platelet parameters**				
PLT, × 10^9^/L, $\overline{x }$ ± *s*	196.19 ± 64.29*	243.47 ± 67.89	− 4.689	< 0.001
MPV, fL, $\overline{x }$ ± *s*	10.50 ± 1.16*	9.58 ± 1.06	5.454	< 0.001
PDW, %, *M* (*P*_25_, *P*_75_)	12.50 (11.38, 14.30)*	15.95 (15.68, 16.10)	− 7.999	< 0.001
**Kidney function parameters**				
Glu, mol/L, *M* (*P*_25_, *P*_75_)	6.10 (5.50, 7.08)*	4.70 (4.10, 5.90)	− 5.978	< 0.001
Cr, μmol/L, $\overline{x }$ ± *s*	65.87 ± 13.85*	57.67 ± 10.26	4.410	< 0.001
Ur, mmol/L, *M* (*P*_25_, *P*_75_)	3.74 (3.13, 4.68)*	4.30 (3.54, 5.15)	− 2.375	0.018
CO_2_-CP, mmol/L, *M* (*P*_25_, *P*_75_)	26.00 (23.28, 27.00)*	26.00 (24.60, 28.25)	− 2.575	0.010
**Liver function parameters**				
TP, g/L, $\overline{x }$ ± *s*	69.45 ± 4.71*	65.72 ± 3.78	5.723	< 0.001
ALB, g/L, $\overline{x }$ ± *s*	40.06 ± 3.59*	39.00 ± 2.95	2.112	0.036
AST, U/L, *M* (*P*_25_, *P*_75_)	23.00 (19.00, 29.00)*	20.50 (15.00, 25.00)	− 3.076	0.002
ALT, U/L, *M* (*P*_25_, *P*_75_)	19.50 (14.00, 32.25)	25.00 (15.75, 41.00)	− 1.233	0.217
ALP, U/L, *M* (*P*_25_, *P*_75_)	68.50 (56.50, 83.00)*	61.00 (51.00, 76.00)	− 1.991	0.046
GGT, U/L, *M* (*P*_25_, *P*_75_)	20.00 (14.75, 37.25)*	28.50 (18.75, 50.25)	− 2.410	0.016
TBIL, μmol/L, *M* (*P*_25_, *P*_75_)	9.80 (7.78, 13.28)	9.75 (7.90, 12.83)	− 0.118	0.906
DBIL, μmol/L, *M* (*P*_25_, *P*_75_)	3.30 (2.50, 4.13)	3.50 (2.68, 4.83)	− 0.887	0.375
IBIL, μmol/L, *M* (*P*_25_, *P*_75_)	6.35 (4.80, 9.33)	6.20 (4.55, 8.50)	− 0.853	0.394
**Electrolyte parameters**				
Na, mmol/L, *M* (*P*_25_, *P*_75_)	140.00 (138.00, 141.00)*	141.00 (139.00, 142.00)	− 2.436	0.015
K, mmol/L, $\overline{x }$ ± *s*	4.07 ± 0.42*	4.34 ± 0.37	− 4.522	< 0.001
Cl, mmol/L, *M* (*P*_25_, *P*_75_)	103.00 (100.75, 105.00)*	106.00 (104.75, 108.00)	− 6.479	< 0.001

*WBC* white blood cell, *NE* neutrophil count, *LY* lymphocyte count, *MO* monocyte count, *EO* Eosinophil, *BA* basophil, *RBC* red blood cell, *HCT* hematocrit, *HGB* hemoglobin, *MCV* mean corpuscular volume, *MCH* mean corpuscular hemoglobin, *MCHC* mean corpuscular hemoglobin concentration, *RDW* red blood cell distribution width, *PLT* platelet count, *MPV* mean platelet volume, *PDW* platelet distribution width, *Glu* Glucose, *Cr* creatinine, *Ur* urea, *CO*_*2*_*-CP* carbon dioxide combining power, *TP* total protein, *ALB* Albumin, *AST* aspartate aminotransferase, *ALT* alanine aminotransferase, *ALP* alkaline phosphatase, *GGT* γ-glutamyl transpeptidase, *TBIL* total bilirubin, *DBIL* direct bilirubin, *IBIL* indirect bilirubin, *Na* sodium, *K* potassium, *Cl* chloride, $\overline{x }$ ± *s* Average ± standard deviation, *M* (*P*_25_, *P*_75_) Median (25th percentile, 75th percentile).

*Significant difference between the two groups, *P* < 0.05.

### Establishing a predictive model for turning negative in patients with moderate COVID-19

**Figure 3:**
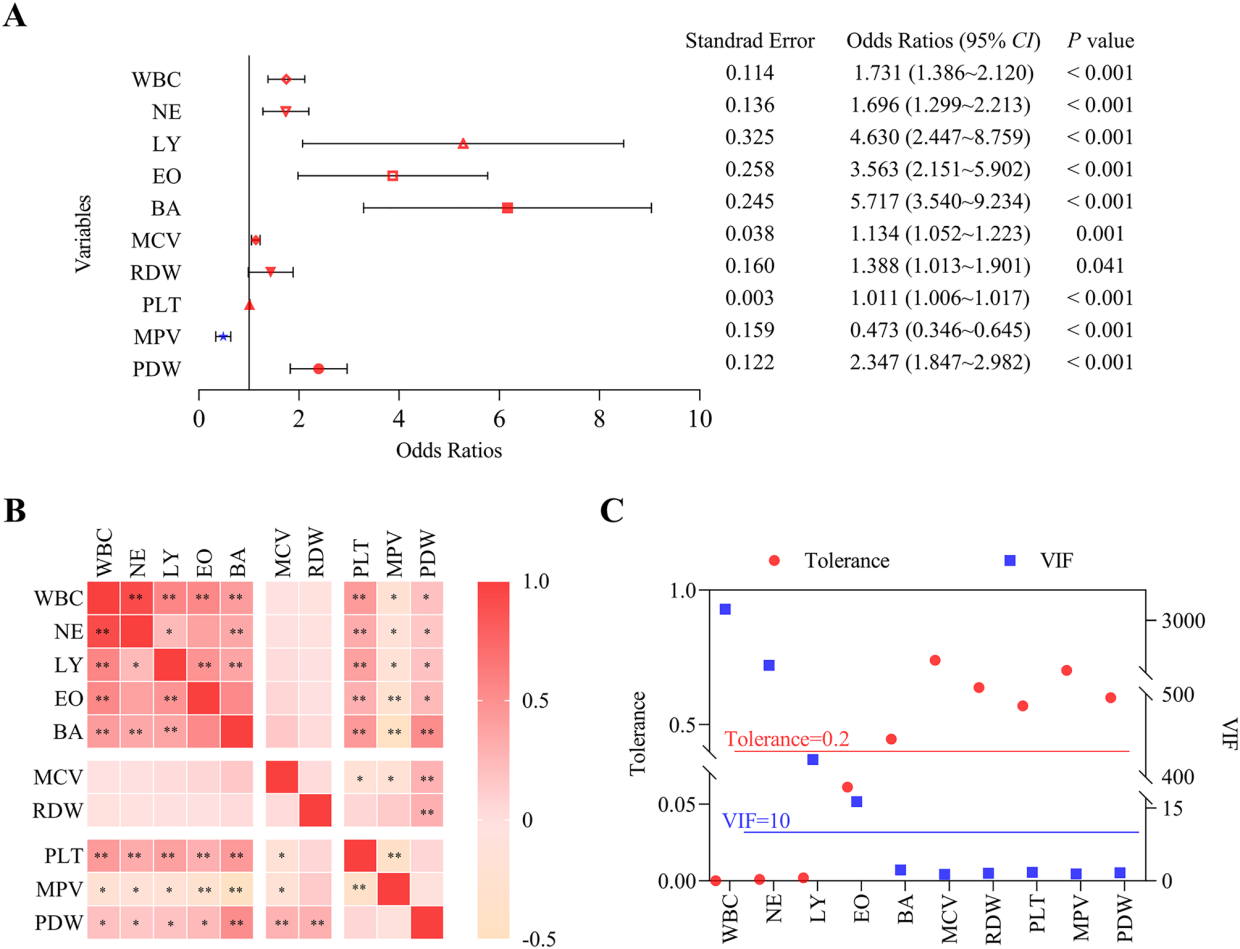
Screening for independent factors associated with patient improvement. (**A**) Forest plot based on univariate logistic regression analysis. (**B**) Correlation heat map of 10 significant difference tests. (**C**) Collinearity diagnostics. *CI* confidence interval, *VIF* variance inflation factor. **P < 0.001, *P < 0.05.

**Figure 4:**
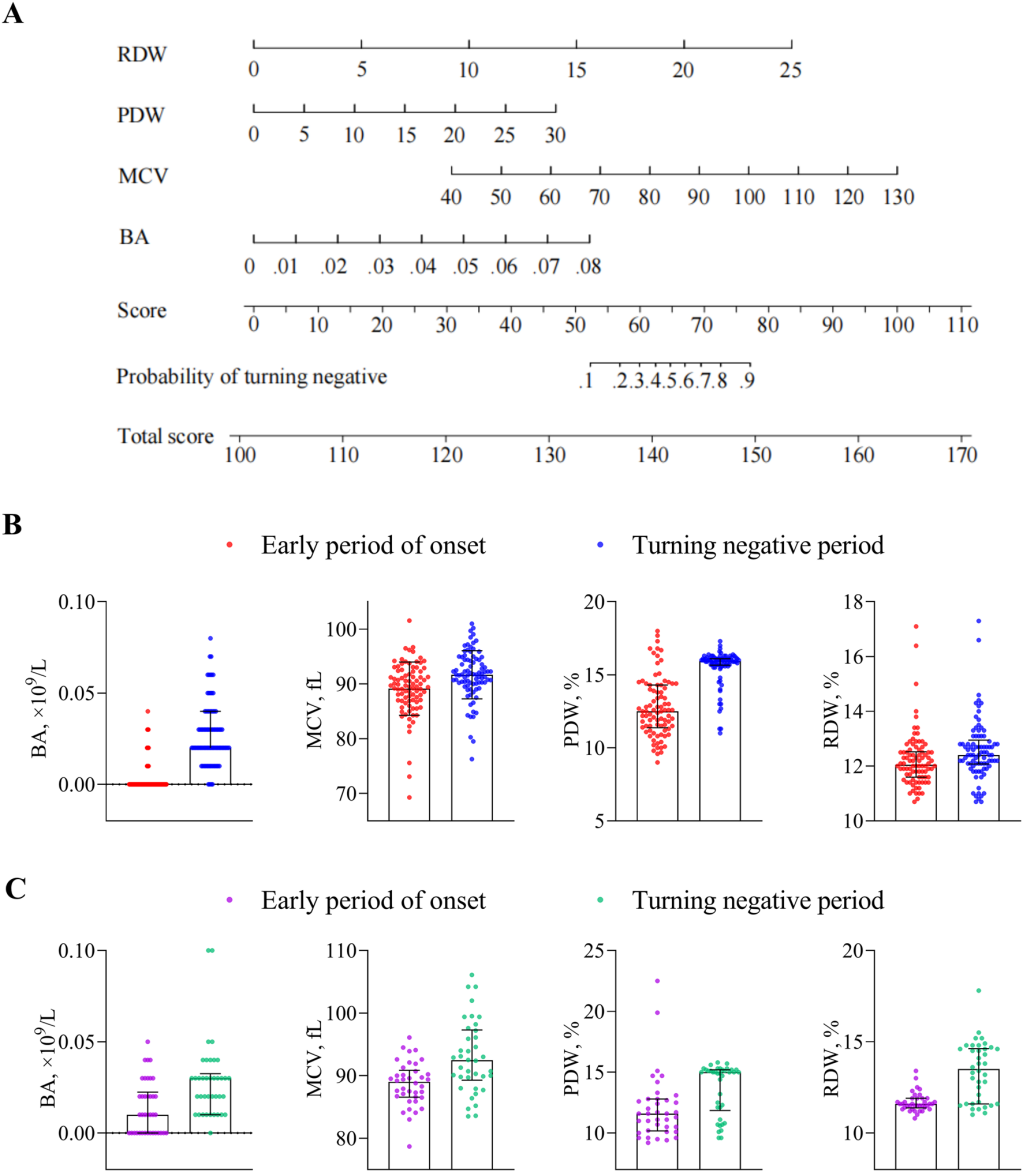
Visual representation of the model. (**A**) Nomogram to illustrate how BA, MCV, RDW, PDW are related to recovery. (**B**) Distribution of BA, MCV, RDW, and PDW in training cohort. (**C**) Distribution of BA, MCV, RDW, and PDW in verification cohort.

We explored the relationship between hematocyte and recovery of patients with moderate COVID-19. The univariate logistic regression analysis showed that at the early onset and turning negative periods, the differences in WBC, NE, LY, EO, BA, MCV, RDW, PLT, MPV, and PDW were statistically significant (*P* < 0.05) (Fig. 3A). The Spearman correlation was performed on the above variables and the results showed significant correlations between most of them (*P* < 0.05) (Fig. 3B). To eliminate redundant indicators and avoid covariance among highly correlated indicators, we performed collinearity diagnostics to screen the variables for subsequent inclusion in the multifactor model. The results showed that WBC, NE, LY, and EO had multicollinearity (VIF > 10 and tolerance < 0.2) (Fig. 3C), so these four variables were excluded from future calculations. BA, MCV, RDW, PLT, MPV, and PDW were included in the multifactor logistic regression and the model was fitted using the Backward: Likelihood Ratio method. The results showed that BA (OR 6.372; 95% CI 3.284–12.363; *P* = 0.001), MCV (OR 1.244; 95% CI 1.088–1.422; *P* < 0.001), RDW (OR 2.585; 95% CI 1.261–5.297; *P* = 0.010), PDW (OR 1.559; 95% CI 1.154–2.108; *P* = 0.004) could jointly predict recovery in patients with moderate COVID-19 (Sensitivity 95.3%, Specificity 91.9%). The combined model was presented in a nomogram. Each variable was assigned a score, and the total score was calculated by summing the individual scores, which reflected the probability of a patient recovering from COVID-19 (Fig. 4A). In both the training and verification cohorts, BA, MCV, RDW, and PDW were lower in the early onset period compared with the turning negative period, and the differences were statistically significant (*P* < 0.05) (Fig. 4B,C).

### Evaluating and validating a predictive model for turning negative in patients with moderate COVID-19

**Figure 5:**
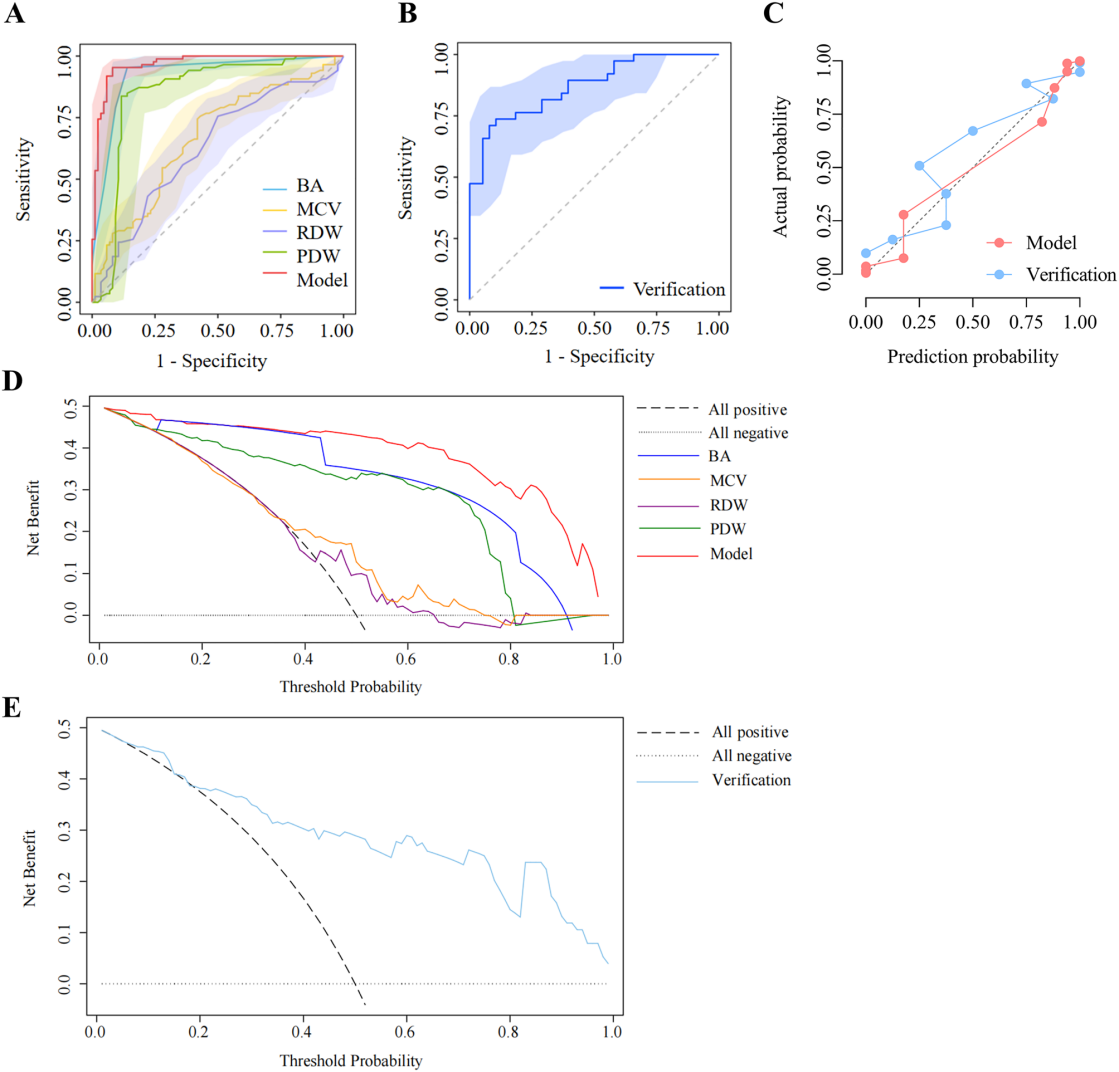
Model performance in the training and verification cohorts. (**A**) Receiver operating characteristic curve of training cohort. (**B**) Receiver operating characteristic curve of verification cohort. (**C**) Calibration curve. (**D**) Decision curve analysis of training cohort; (**E**) decision curve analysis of verification cohort. Model, Combined model of basophil, mean corpuscular volume, red blood cell distribution width and platelet distribution width; the shaded parts represented the 95% confidence interval of the areas under receiver operating characteristic curves.

The ROC curves showed that the combined model had better discrimination compared with any single variable model in the training cohort (AUC = 0.968; 95% CI 0.943–0.992; *P* < 0.001) (Fig. 5A) and in the external verification cohort (AUC = 0.870; 95% CI 0.793–0.948; *P* < 0.001) (Fig. 5B). The detailed parameters of the ROC curves were shown in Table 3. The Hosmer–Lemeshow goodness-of-fit showed results in the calibration curve for the training cohort (χ^2^ = 8.804; *P* = 0.359) and the verification cohort (χ^2^ = 7.502, *P* = 0.484) (Fig. 5C). The DCA showed that the net benefit of the combined model was significantly higher than that of an arbitrary single variable model in the training and verification cohorts (Fig. 5D,E).

**Table 3:** Characteristics of the receiver operating characteristic curve.

Variables	AUC (95% *CI*)	Sensitivity (95% *CI*)	Specificity (95% *CI*)	Youden index	*P* value
BA	0.925 (0.882–0.968)	0.953 (0.885–0.987)	0.860 (0.769–0.926)	0.814	< 0.001
MCV	0.669 (0.588–0.750)	0.756 (0.651–0.842)	0.570 (0.458–0.676)	0.326	< 0.001
RDW	0.629 (0.545–0.712)	0.756 (0.651–0.842)	0.500 (0.390–0.610)	0.256	0.004
PDW	0.853 (0.787–0.918)	0.837 (0.742–0.908)	0.884 (0.797–0.943)	0.721	< 0.001
Model	0.968 (0.943–0.992)	0.953 (0.885–0.987)	0.919 (0.839–0.967)	0.872	< 0.001
Verification	0.870 (0.793–0.948)	0.737 (0.569–0.866)	0.895 (0.752–0.971)	0.632	< 0.001

## Discussion

This study had the following innovative findings: (1) This study used a variety of statistical methods to screen independent factors, including univariate logistic regression, Spearman correlation, collinearity diagnosis, and Backward: Likelihood Ratio method, which were conducive to fitting a more efficient prediction model. (2) This study evaluated the discrimination, calibration, and clinical usefulness of the model using training and external validation cohorts, which could more comprehensively demonstrate the prediction ability of the model based on complete blood count. (3) This study revealed that BA, MCV, RDW, and PDW could be used to predict the recovery of patients with moderate COVID-19. It was worth noting that although the medians of BA, MCV, RDW, and PDW were within the normal ranges at both admission and discharge, these values were higher at discharge. Thus, the “elevation” described in this study was not an abnormal increase outside the reference range.

BA were rare blood leukocytes produced by bone marrow progenitor cells (approximately 2%). Conceição-Silva et al. showed that they had extracellular traps with a fungicidal and antifungal activity that might play a protective role during COVID-19 infection^23^. Rodriguez et al. discovered that BA in patients with severe COVID-19 increased significantly from the acute phase to the recovery phase^24^. Our findings were consistent with the above studies and we concluded that elevated BA predicted improvement and could be a prognostic marker for recovery in patients with moderate COVID-19. However, other studies suggested that a progressive increase in BA was a risk factor for COVID-19 lethality, which contradicted our findings^25^. MCV and RDW were used as parameters to assess the mean volume and size heterogeneity of erythrocytes. Our study showed no significant changes in erythrocyte morphology in patients with moderate COVID-19. A slight increase in MCV and RDW within the normal range could predict improvement in patients. Studies concluded that the uneven red blood cell distribution was closely related to the poor prognosis and mortality of COVID-19, but some studies believed that there was no significant correlation between them^26–28^. These conflicting views suggested the need for future in-depth investigations. The concept of PDW was like RDW and reflected the heterogeneity of platelet size. Wang et al. found that PDW was significantly higher in patients with mild COVID-19 at discharge compared with at admission and that PDW had a potential diagnostic value for mild COVID-19^29^. Our findings were consistent with these results and demonstrated that elevated PDW could be used to predict recovery in patients with moderate COVID-19. In contrast, Bommenahalli Gowda et al. showed that elevated PDW was significantly associated with increased mortality in COVID-19^30^. Possible reasons for the differences in study results included the following: our study used moderate cases only, patients with different subtypes were excluded, and it was conducted in Jilin province, China, where the COVID-19 severity was relatively low.

This study had several limitations: (1) This study had a small sample size with only 86 patients included, which might have affected the statistical power. (2) This study was a retrospective study, we lacked the results of some laboratory indicators, thus failing to show the changes in all laboratory indicators at the time of admission and discharge. (3) Considering the advantages of CBC in terms of simplicity, speed, and cost-saving, this study built a prediction model for CBC indicators only, without incorporating other indicators that may have better prediction performance. (4) Changes in these parameters might have been influenced by medication, but due to the limitations of retrospective studies, we were unable to intervene in the patients’ medication use. (5) The chest X-ray and computed tomography scan results of the patients were not collected in this study, so it was not possible to analyze the influence of the image features on the rehabilitation of the patients.

## Conclusion

This study developed and validated a reliable nomogram model for predicting the recovery in patients with moderate COVID-19. We concluded that small elevations in BA, MCV, RDW, and PDW within the normal ranges could jointly predict disease progression in patients with moderate COVID-19 and help clinicians to better monitor disease progression in these patients.

## Data Availability

The datasets used and/or analysed during the current study are available from the corresponding author on reasonable request.
